# Endocytic Mechanism of Internalization of Dietary Peptide Lunasin into Macrophages in Inflammatory Condition Associated with Cardiovascular Disease

**DOI:** 10.1371/journal.pone.0072115

**Published:** 2013-09-05

**Authors:** Anthony Cam, Mayandi Sivaguru, Elvira Gonzalez de Mejia

**Affiliations:** 1 Department of Food Science and Human Nutrition, University of Illinois at Urbana-Champaign, Urbana, Illinois, United States of America; 2 Institute for Genomic Biology, University of Illinois at Urbana-Champaign, Urbana, Illinois, United States of America; King's College London School of Medicine, United Kingdom

## Abstract

Cardiovascular disease (CVD) is the leading cause of death in the United States. Diet influences risk factors associated with CVD and atherosclerosis, a major vascular disease that arises from inflammation. Lunasin, a peptide derived from plant foods such as soybeans, contains a unique Arg-Gly-Asp cell-adhesion motif and inhibits the pathways involved in the inflammatory cascade. The objective was to determine the mechanism by which lunasin is internalized into human THP-1 macrophages, investigate the expression of endocytic membrane proteins in inflammatory conditions and to identify the pathways involved. While lipopolysaccharide (10 nM), vitronectin (130 nM) and a combination of these two molecules enhanced lunasin uptake and increased basal αVβ3 integrin expression, lunasin reduced αVβ3 expression by 25.5, 26.8 and 49.2%, respectively. The pretreatment of cells with brefeldin A (71 µM), an inhibitor of protein trafficking, inhibited lunasin internalization by up to 99.8%. Lunasin increased caveolin-1 expression by up to 204.8%, but did not modulate clathrin. The pretreatment of macrophages with nystatin (54 µM), an inhibitor of caveolae-dependent endocytosis, reduced lunasin internalization. The presence of amantadine (1 mM) and amiloride (1 mM), inhibitors of clathrin-mediated endocytosis and macropinocytosis, abolished lunasin cell entry. Lunasin elicited a transient reduction in intracellular levels of Ca^2+^ in LPS-induced macrophages. The results suggest that internalization of lunasin into macrophages is amplified in inflammatory conditions and is primarily mediated by endocytic mechanisms that involve integrin signaling, clathrin-coated structures and macropinosomes. Lunasin may be responsible for attenuation of CVD risk factors by interacting with pathways involved in endocytosis and inflammation.

## Introduction

Cardiovascular disease (CVD) is the leading cause of human death in the United States, and inflammation is directly involved in the initiation and progression of atherosclerotic lesions [1]. CVD was responsible for 1 out of every 3 human deaths in the U.S. in 2009, and approximately one American will die of a coronary event every minute [2]. Diet substantially impacts the risk factors, such as hypercholestolemia, hypertension, diabetes and obesity, all of which are highly associated with the development of CVD and atherosclerosis. Therefore, research that is designed to identify and elucidate the effects of dietary bioactive compounds, such as lunasin, that possess the potential to mitigate inflammatory states and atherosclerosis, would provide knowledge that could be used to augment current efforts at reducing the prevalence of CVD. Furthermore, the characterization of the intracellular structures and effectors involved in mediating the endocytosis of naturally-occurring constituents of human macrophages would provide insight into the potential molecular targets of dietary compounds with biological activity and the mechanisms by which they ameliorate the risk factors of CVD.

Lunasin is a 43-amino acid peptide that was originally isolated from soybeans and contains a unique Arg-Gly-Asp (RGD) cell-adhesion motif that is responsible for its bioactive properties [3]. Lunasin has been demonstrated to be bioavailable in humans after the consumption of soy protein foods and biologically active in tissues from rats [4], [5]. Chronic inflammation induces the aggregation of macrophages that highly express αVβ3 integrins to atherosclerotic lesions, and this receptor subsequently induces the release of inflammatory cytokines [6]. Lunasin has been reported to inhibit αVβ3 integrin-mediated pro-inflammatory markers and to downregulate the Akt-mediated NF-κB pathways through its interaction with the αVβ3 integrin [7]. Furthermore, the interaction of lunasin with the integrin RGD-receptors at the cellular membrane have been associated with its anti-inflammatory properties [7].

The cellular internalization of RGD peptides is primarily mediated by the clathrin, caveolae and macropinocytosis endocytic pathways at the plasma membrane [8]. As one of the primary effectors of endocytic transport at the plasma membrane, clathrin-mediated endocytosis is involved in the transport of large extracellular particles into the cell through the receptor-dependent endocytosis of ligands [9]. An alternative route for peptide internalization is through caveolae-mediated endocytosis. Internalization through this pathway is facilitated by lipid rafts in the cell membrane; these rafts contain caveolin-1 proteins that form endosomes, which are then transported throughout the cell [10]. In contrast, macropinocytosis involves the fluid-phase endocytosis of small extracellular particles into the cell [11]. It has been demonstrated that the αVβ3 integrin can be internalized through both the clathrin and caveolae-dependent endocytic pathways as part of the regulation of integrin turnover [12].

The current hypothesis states that lunasin attenuates the αVβ3 integrin expression that is enhanced during inflammation and is internalized into macrophages via integrin-mediated endocytic pathways. Therefore, the objective of this study was to define, *in vitro*, the internalization mechanism of lunasin into human macrophages in inflammatory conditions using fluorescence confocal microscopy. In addition, this study investigated the effect of lunasin on the expression of endocytic membrane proteins and identified the functional pathways that are critical in mediating its internalization by macrophages. Our findings revealed that lunasin, a dietary peptide, attenuated integrin expression enhanced during inflammation, and internalized into cells via integrin-mediated and clathrin-dependent endocytosis.

## Materials and Methods

### Materials

The human acute monocytic leukemia cell line (THP-1) and the Roswell Park Memorial Institute-1640 media (RPMI-1640) were purchased from the American Type Culture Collection (Manassas, VA, USA). The fetal bovine serum (FBS) was purchased from Thermo Scientific (Logan, UT, USA). The streptomycin/penicillin and sodium pyruvate were purchased from Cellgro (Manassas, VA, USA). The LPS from *Escherichia coli* O55: B5 and the phorbol 12-myristate 13-acetate (PMA) were purchased from Sigma-Aldrich (St. Louis MO, USA). The human recombinant vitronectin was purchased from LD Biopharma Inc. (San Diego, CA, USA). The mouse antibody to αVβ3 integrin was purchased from Santa Cruz Biotechnology (Santa Cruz, CA, USA). The Alexa Fluor 568 Goat Anti-Rabbit IgG, Alexa Fluor 488 Goat Anti-Mouse IgG, Fluo-4, AM, cell permeant, Image-iT FX signal Enhancer, ProLong Gold antifade reagent with DAPI, and phenol red-free RPMI-1640 were purchased from Life Technologies (Carlsbad, CA, USA). The paraformaldehyde (16%) aqueous solution was purchased from Electron Microscopy Sciences (Hatfield, PA, USA). The IbiTreat µ-Slide 8-well microscopy chambers and the ibiTreat μ-Dish 35 mm-high walls were purchased from ibidi (Munich, Germany). All of the other chemicals were purchased from Sigma-Aldrich (St. Louis, MO, USA), unless otherwise stated. Lunasin was purified (>95%) in our laboratory from defatted soybean flour (7).

### Cell Culture

#### Differentiation of THP-1 monocytes

THP-1 monocytes were cultured as described in our previously published procedure (7).

### Cell Proliferation Assay

The cell proliferation assay was performed with THP-1 macrophages (7). Treatment with up to 100 µM lunasin was not cytotoxic.

### Treatment of THP-1 macrophages and immunostaining

Treatment of the THP-1 macrophages and immunostaining were performed as described in our previously published procedure (7). Images were taken of a monolayer of at least 5 cells for four independent fields per well for three independent replicates.

### Confocal laser-scanning microscopy and two-dimensional rendering of the lunasin-treated THP-1 macrophages

Samples were imaged using a 63×/1.4 Oil DIC M27 objective with a Zeiss LSM 700 laser-scanning confocal microscope (Carl Zeiss AG, Germany). The images were obtained using a 488 Ar (10 mW) laser line for αVβ3 integrin or clathrin (500–550 nm emission), 555 nm (10 mW) laser line for lunasin or caveolin-1 (600–650 nm) and 639 nm (5 mW) laser line for αVβ3 integrin (650–700 nm emission) in order to perform triple labeling, and a differential interference contrast image was obtained using the transmitted-light configuration of the same 633 nm laser using a TPMT module. The individual channels were obtained using a sequential scanning mode to prevent bleed-through of the excitation signal. Laser power, gain and offset were kept constant across the samples, and the samples were scanned in a high-resolution format of 1024×1024 pixels with 2/4 frame averaging. Multiple two-dimensional (2D) image stacks (512−512 pixels) were taken along the Z axis. Single optical planes of the individual channels for lunasin, αVβ3 integrin, clathrin and caveolin-1 were captured (with or without zoom) and all of the optical planes were displayed as a gallery. The expression and area sums (µm^2^) of the raw images were quantified with AxioVision Rel 4.8 (Carl Zeiss AG, Germany). All of the image panels were resized and consolidated using the GNU Image Manipulation Program, and the brightness of the final collage of images displayed was increased by 40% as a whole.

### Live-cell imaging of intracellular Ca^2+^ in macrophages

Differentiated THP-1 macrophages were incubated with 10 µM Fluo-4 AM in 0.01% DMSO for 30 min at 37°C in serum and phenol red-free RPMI-1640 media. Fluo-4 labeled macrophages were washed once with fresh serum-free RPMI-1640 media (10% FBS). Treatments were added at Frame 100, and images were captured in frames per second and a 1.27 µs pixel dwell time. Movies of the intracellular calcium levels for the key experiments were made in the Zen 2010 software as stated above, using the pseudocolor (rainbow) range indicator lookup table (LUT), in which blue and red correspond to the lowest and highest intensities, respectively.

### Statistical Analysis

Data were analyzed using a one-way analysis of variance (ANOVA) using Statistical Analysis System software version 9.3 (SAS Institute, Cary, NC, USA). Means of n = 3 were generated and adjusted with Least Significant Difference. Significant differences were reported at p<0.05.

## Results

### Lunasin attenuated the αVβ3 integrin membrane expression that is initially upregulated by inflammatory ligands

**Figure 1:**
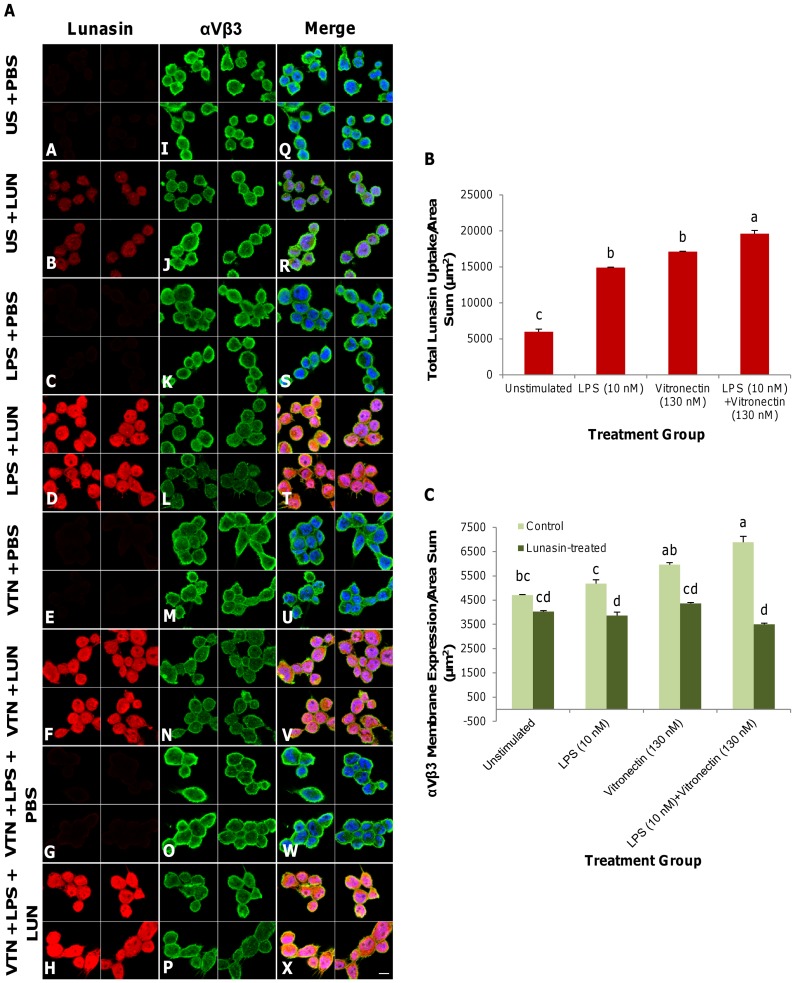
Lunasin reduced αVβ3 integrin membrane expression initially upregulated by inflammatory ligands. Two-dimensional optical planes demonstrate the immunocytochemical localization of lunasin (red), αVβ3 integrin (green) and nuclei (blue) (**Figure 1A**). Unstimulated macrophages or macrophages have been induced with LPS (10 nM), vitronectin (130 nM) or the two molecules in combination for 24 h and treated with lunasin (1 µM) for 1 h. Compared to the PBS control (**A–B**), lunasin uptake increased sequentially upon stimulation with LPS (**C–D**), vitronectin (**E–F**), or both molecules in combination (**G–H**). Lunasin (1 µM) treatment reduced the basal expression level of αVβ3 integrin at the cellular membrane (**I–J**). The upregulation of the total basal expression level of αVβ3 integrin at the membrane in the presence of LPS (**K–L**), vitronectin (**M–N**) or both molecules in combination (**O–P**) was subsequently attenuated by lunasin treatment (1 µM). The merged image (with nuclei) shows increased lunasin uptake and a concomitant reduction in the level of αVβ3 integrin expression at the surface of the plasma membrane (**Q–X**), as well as their respective expression levels (**Figure 1B, 1C**). Four independent fields of view from three independent cellular replicates were merged together per treatment group. In Figure 1B and 1C, the data represent the average of triplicates or quadruplicates ± SE. Means with different letters are significantly different from each other (n = 3, p<0.05). US  =  unstimulated, PBS  =  phosphate-buffered saline, LPS  =  lipopolysaccharide (10 nM), VTN  =  vitronectin (130 nM), LUN  =  lunasin (1 µM). Bar  = 10 µm.

**Figure 1A** **, panels A–H**, depicts the results of two-dimensional immunocytochemical fluorescence confocal microscopy performed on THP-1 human macrophages that have been induced with the pro-inflammatory agents LPS (10 nM), vitronectin (130 nM) or a combination of these molecules; all of the conditions resulted in increased lunasin uptake. Lunasin internalization was markedly higher in the stimulated groups, and lunasin was most readily endocytosed when the cells were exposed to a combination of LPS and vitronectin. Compared to the PBS-treated and unstimulated control, lunasin uptake increased 2.5-, 2.9- and 3.3-fold in the LPS-, vitronectin- and combination-stimulated groups, respectively (**Figure 1B**). Compared to the control condition (**Figure 1A:** **I**), the total basal expression of the αVβ3 integrin (**Figure 1A:** **K, M, O**) in the membrane increased upon induction with LPS, vitronectin or the two molecules in combination. Lunasin treatment attenuated the basal expression of the αVβ3 integrin at the surface of the plasma membrane (**Figure 1A**: **J, L, N, P**), reducing it by 1.2-, 1.3-, 1.4- and 2.0-fold relative to the untreated controls (p<0.05) (**Figure 1A:** **Q–X**, **Figure 1C**).

### Intracellular accumulation of lunasin is associated within clathrin-coated structures (CCS)

**Figure 2:**
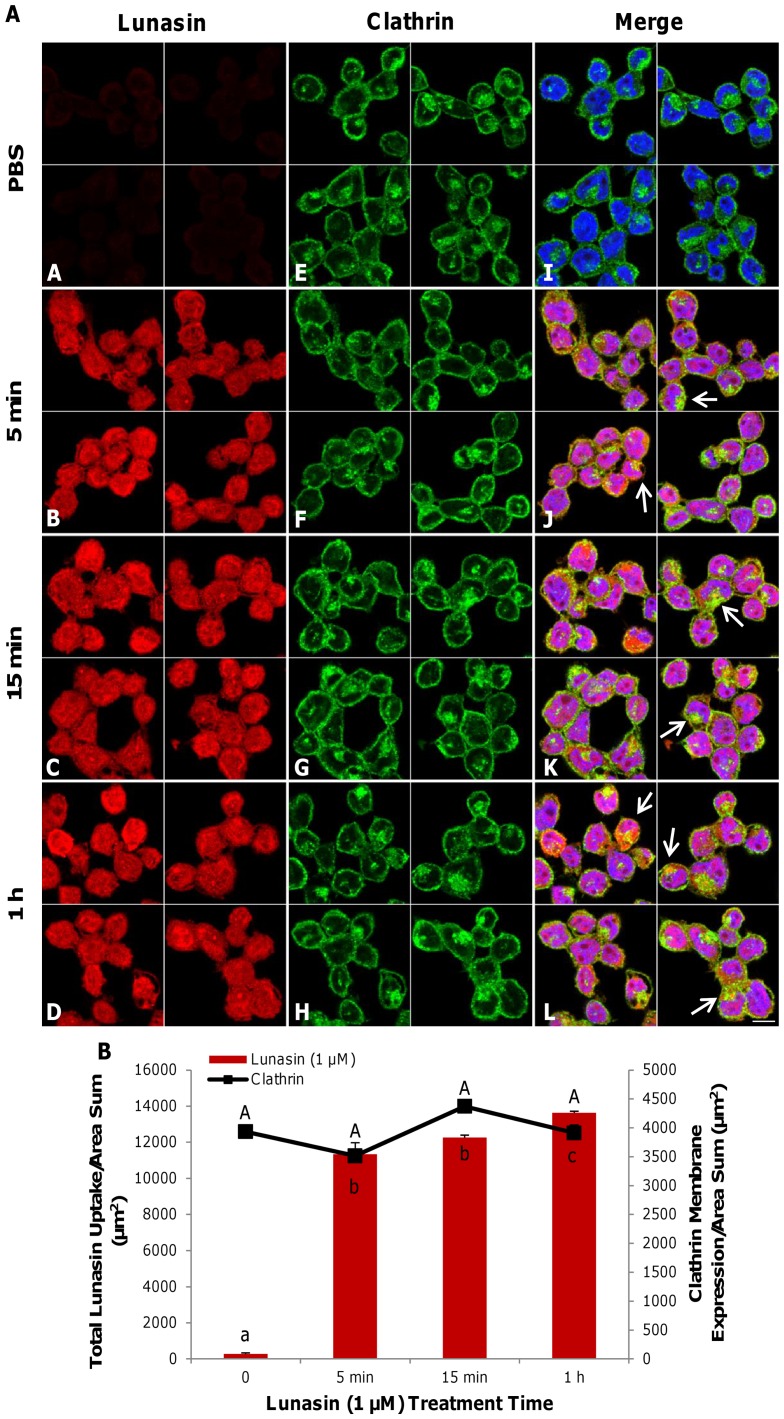
Lunasin accumulated within intracellular clathrin-coated structures. Two-dimensional optical planes showing the immunofluorescent localization of lunasin (red), clathrin (green) and nuclei (blue) (**Figure 2A**). Total lunasin uptake by macrophages increased over time (**A–D**), while clathrin expression at the membrane surface was unchanged by the treatment (**E–H**). Immunofluorescence and the merged image of lunasin, clathrin and nuclei are shown (**I–L**). Lunasin localized to specific regions with high clathrin expression (**J–L**, arrows). Quantification of total lunasin uptake and clathrin membrane expression over the time-course of lunasin treatment (**Figure 2B**). Four independent fields of view from three independent replicates of cells were merged together per treatment group. In Figure 2B, the data represent the average of triplicates or quadruplicates ± SE. Means with different letters are significantly different from each other (n = 3, p<0.05). Bar  = 10 µm.

Immunofluorescent analysis of two-dimensional median optical planes of lunasin-treated (1 µM) macrophages demonstrated that lunasin uptake increased over time, although the values were only statistically different after 1 h of exposure (**Figure 2A:** **A–D**). Lunasin did not modulate the expression of clathrin at the surface of the cell membrane over time (**Figure 2A:** **E–H**). Lunasin was internalized in intracellular vesicles and localized inside large aggregates of CCS (**Figure 2A:** **J, K, L; arrows, Video S1**). The quantification of the total intracellular lunasin uptake and clathrin expression at the membrane indicated that clathrin levels remained unaffected over time (**Figure 2B**).

### Clathrin expression at the plasma membrane remained constitutive in stimulated macrophages upon lunasin treatment

**Figure 3:**
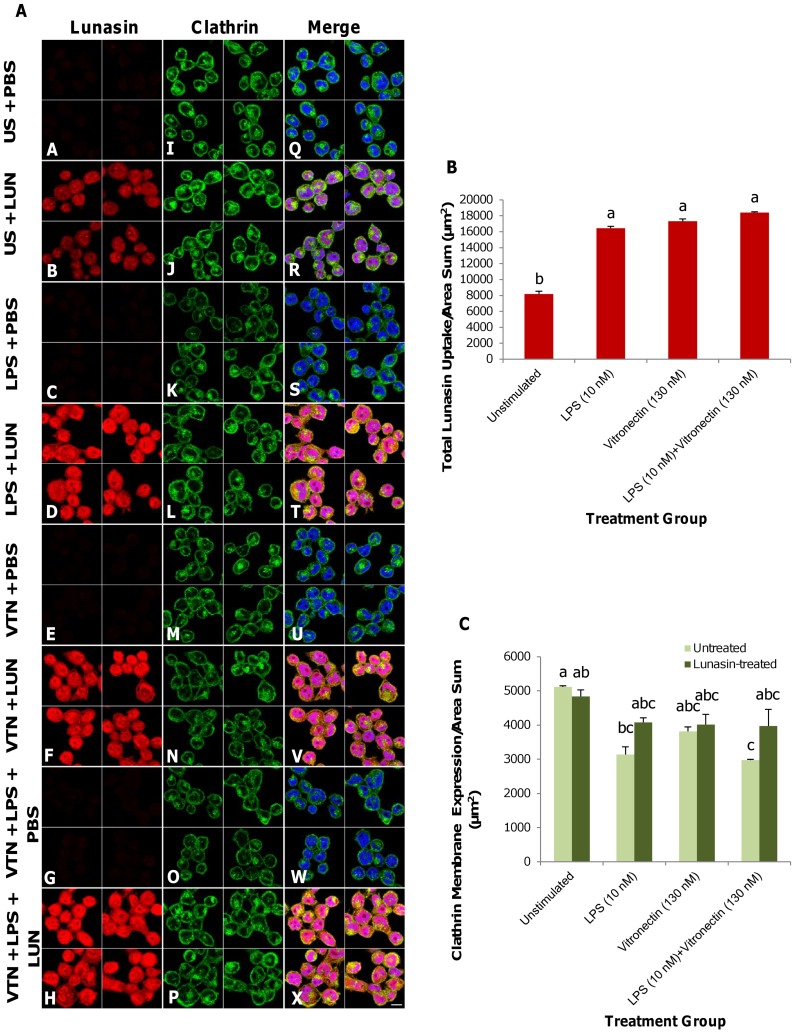
Clathrin remained constitutively expressed in stimulated macrophages treated with lunasin. Two-dimensional median optical planes demonstrating immunocytochemical localization of lunasin (red), clathrin (green) and nuclei (blue) (**Figure 3A**). Unstimulated macrophages or macrophages have been induced with LPS (10 nM), vitronectin (130 nM) or both molecules in combination for 24 h, and treated with lunasin (1 µM) for 1 h. Compared to the PBS control (**A–B**), lunasin uptake increased upon stimulation with LPS (**C–D**), vitronectin (E–F), or both molecules in combination (**G–H**). Induction by LPS or both molecules in combination reduced basal expression levels of clathrin at the membrane, but clathrin expression remained unaffected following lunasin treatment in all groups (**I–P**). Merge image (with nuclei) showing the increased lunasin uptake and the effect of clathrin at the plasma membrane surface (**Q–X**), and their respective expression levels (**Figure 3B, 3C**). Four independent fields of view from three independent replicates of cells were merged together per treatment group. In Figure 3B and 3C, data represent the average of triplicates or quadruplicates ± SE. Means with different letters are significantly different from each other (n = 3, p<0.05). US  =  unstimulated, PBS  =  phosphate buffer saline, LPS  =  lipopolysaccharide (10 nM), VTN  =  vitronectin (130 nM), LUN  =  lunasin (1 µM). Bar  = 10 µm.

Two-dimensional median optical planes illustrating the immunocytochemical localization of lunasin in macrophages that had been induced with LPS (10 nM), vitronectin (130 nM) or the two molecules in combination revealed that induction facilitated the internalization of lunasin, compared to the PBS control (**Figure 3A:** **A–H**). Induction with LPS and LPS and vitronectin in combination decreased the basal expression of clathrin at the membrane by 38.6 and 41.8%, respectively, while vitronectin alone did not exhibit a significant effect relative to the control (**Figure 3A:** **I, K, M, O**). The expression of clathrin at the membrane remained unaffected among all of the groups after the treatment with lunasin (**Figure 3A:** **J, L, N, P**). The results of merging all of the channels (**Figure 3A:** **Q–X**) and quantifying their respective expression levels indicated that the total lunasin uptake did not modulate clathrin expression at the surface of the plasma membrane (**Figure 3B, 3C**).

### Lunasin promoted the recruitment of caveolin-1 to the plasma membrane surface

**Figure 4:**
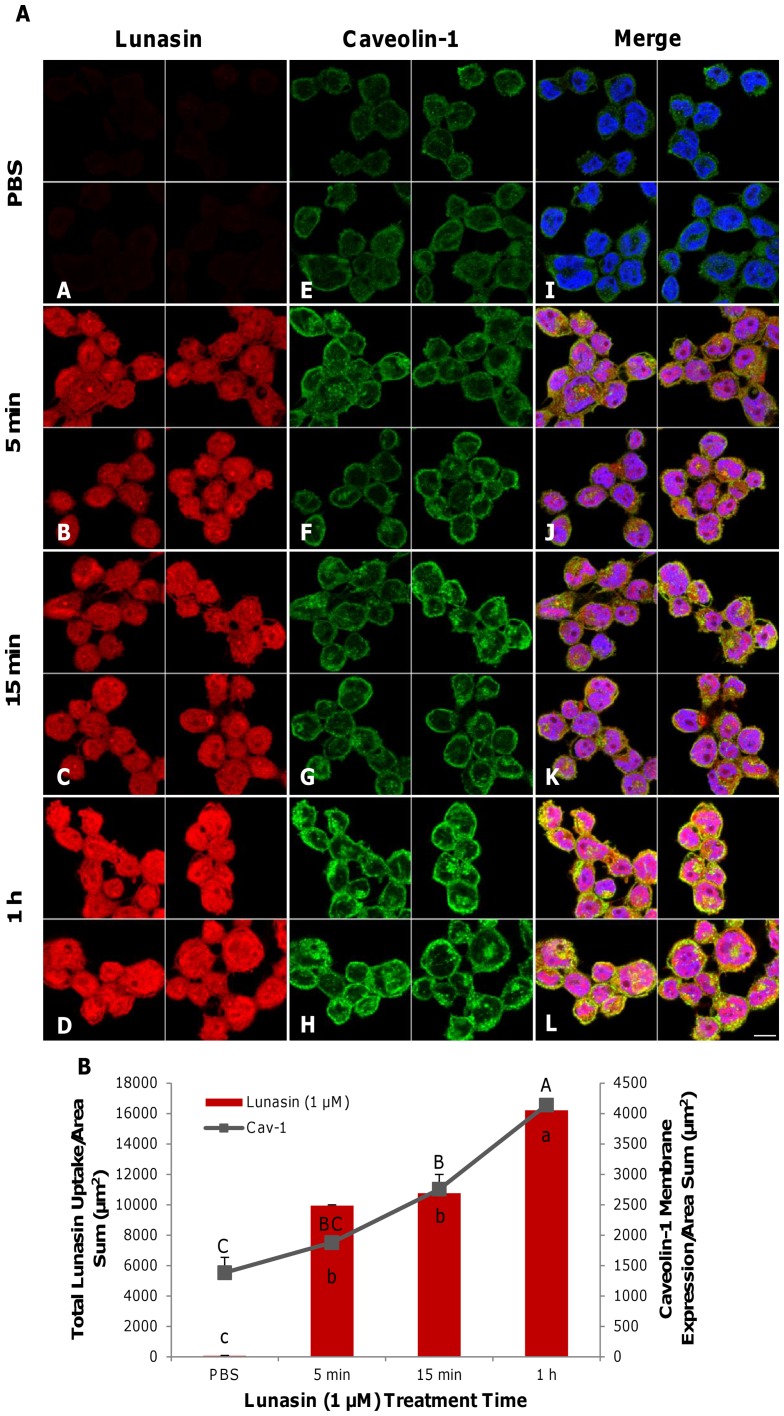
Lunasin promoted the translocation of caveolin-1 to the plasma membrane. Two-dimensional median optical planes showing immunofluorescent localization of lunasin (red), caveolin-1 (green) and nuclei (blue) (**Figure 4A**). Total lunasin uptake by macrophages increased over time (**A–D**) and treatment upregulated the expression of caveolin-1 at the surface of the plasma membrane (**E–H**). Immunofluorescence and merged image for lunasin, caveolin-1 and nuclei are shown (**I–L**). Quantification of the total lunasin uptake and expression of caveolin-1 at the membrane over the time-course of the lunasin treatment (**Figure 4B**). Four independent fields of view from three independent replicates of cells were merged together per treatment group. In Figure 4B, data represent the average of triplicates or quadruplicates ± SE. Capital letters denote statistical values of caveolin-1 expression, whereas lower-case letters represent the statistics for the uptake of lunasin. Means with different letters are significantly different from each other (n = 3, p<0.05). Bar  = 10 µm.

Immunofluorescent analysis of two-dimensional median optical planes of lunasin-treated (1 µM) macrophages revealed that lunasin uptake increased as treatment time progressed, up to 1 h (**Figure 4A:** **A–D**). Lunasin upregulated expression of caveolin-1 at the membrane by 1.4-, 2.0- and 3.0-fold over 5 min, 15 min and 1 h (p<0.05), respectively (**Figure 4A:** **E–H**). The results of merging all of the channels (**Figure 4A:** **I–L)** and quantifying their expression levels revealed that caveolin-1 recruitment to the cell membrane occurred primarily between 15 min and 1 h after lunasin treatment (**Figure 4B**).

### Brefeldin A (BFA) treatment abolished lunasin endocytosis in human macrophages

**Figure 5:**
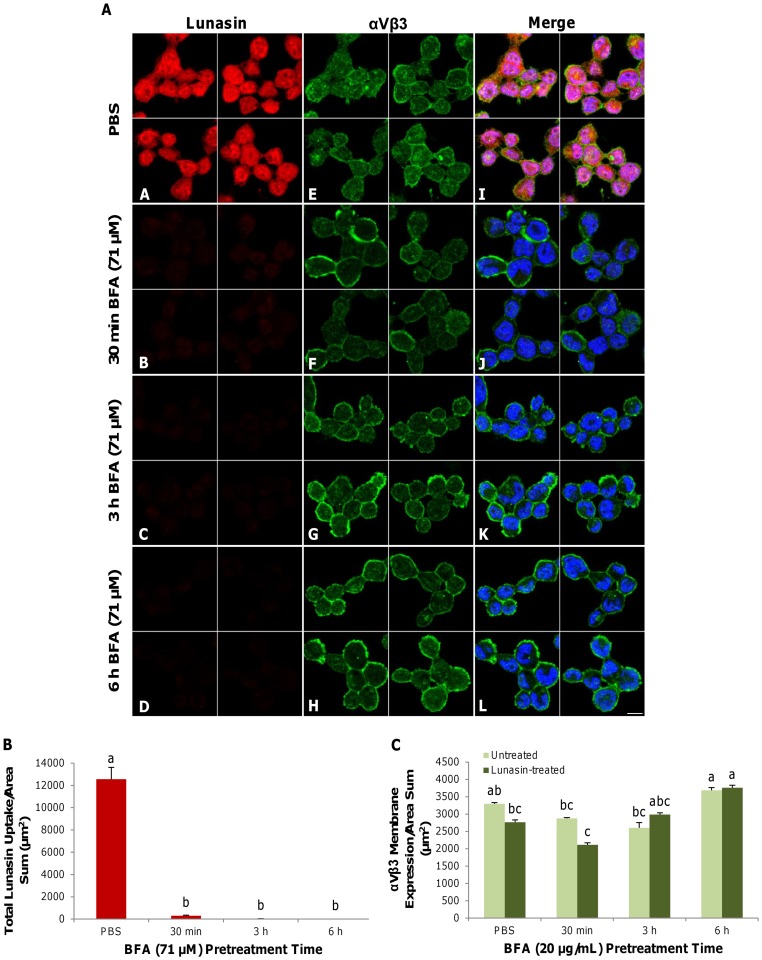
Brefeldin A (BFA) inhibited lunasin internalization within macrophages. Two-dimensional optical planes demonstrating immunocytochemical localization of lunasin (red), αVβ3 integrin (green) and nuclei (blue) (**Figure 5A**). Macrophages were pretreated with BFA (71 µM) for up to 6 h. BFA pretreatment inhibited lunasin internalization over all of the time points (**B–D**). Compared to the PBS control (**E**), lunasin treatment (1 µM) reduced the expression of αVβ3 integrin at the membrane after 30 min of BFA pretreatment (**F**), but αVβ3 integrin expression remained unaffected at all other time points (**G–H**). Merged image (with nuclei) showing the effect of lunasin uptake and αVβ3 integrin expression on BFA pretreatment (**I–L**), and their respective quantified expressions (**Figure 5B, 5C**). In Figure 5B and 5C, data represent the average of triplicates or quadruplicates ± SE. Four independent fields of view from three independent replicates of cells were merged together per treatment group. Means with different letters are significantly different from each other (n = 3, p<0.05). Bar  = 10 µm.

Figure 5A: A–D depicts two-dimensional median optical planes demonstrating the immunocytochemical localization of lunasin in macrophages that had been pretreated with brefeldin A (BFA, 71 µM). BFA pretreatment at 30 min, 3 h and 6 h inhibited lunasin internalization by 97.5, 99.6 and 99.8%, respectively (p<0.05). Following the BFA pretreatment (30 min), lunasin reduced the expression of the αVβ3 integrin at the membrane, but expression of αVβ3 integrin at all other time points remained unaffected (**Figure 5A:** **E–H**). The results of merging all of the channels (**Figure 5A:** **I–L**) and the quantification of the intracellular space and membrane indicated that BFA markedly inhibited the endocytosis of lunasin (**Figure 5B** **, Video S2**). Additionally, the αVβ3 integrin remained essentially constitutively expressed at the membrane over time (**Figure 5C**).

### Lunasin endocytosis is primarily mediated through clathrin-coated structures and macropinosomes

**Figure 6:**
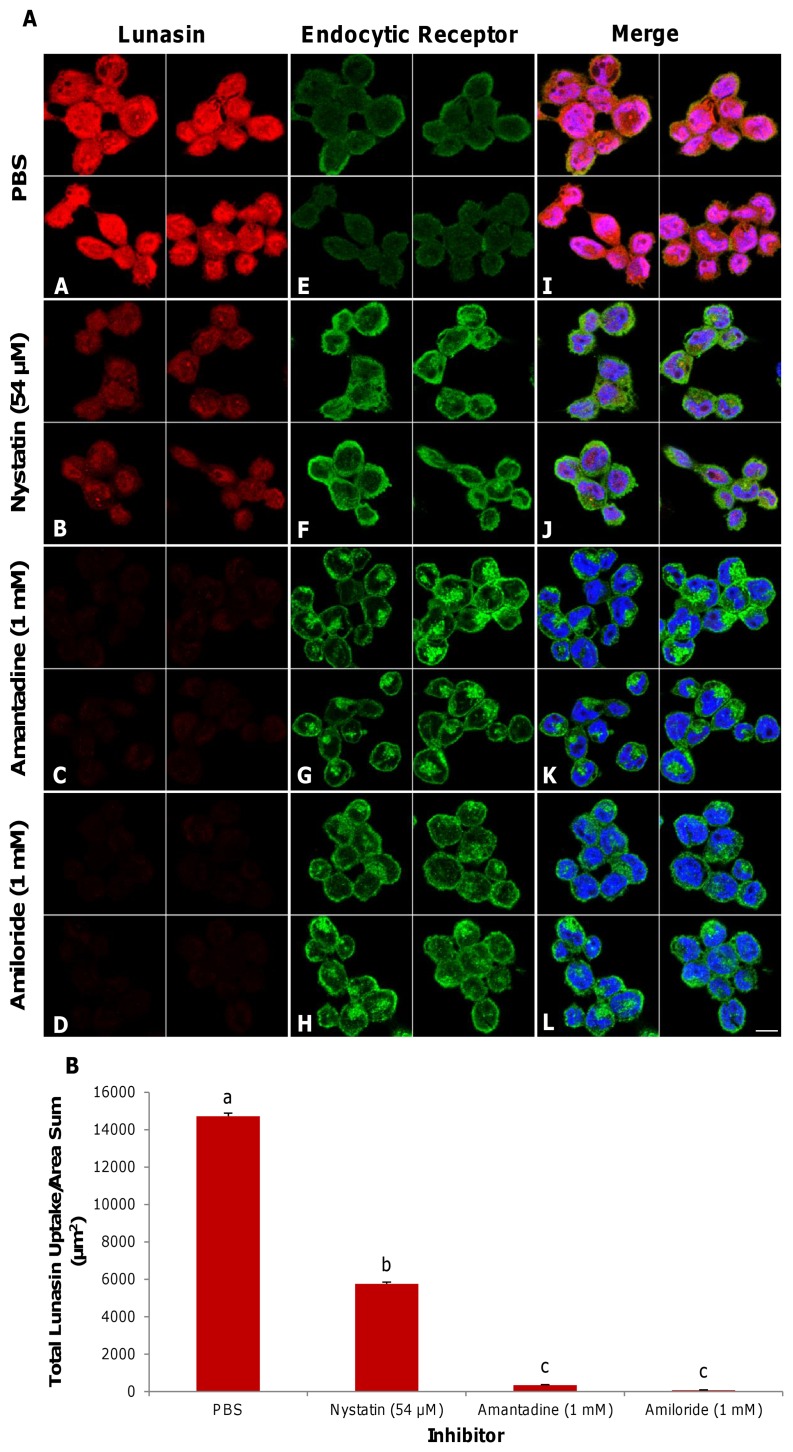
Lunasin internalization is primarily facilitated through clathrin-coated structures and macropinosomes. Two-dimensional immunocytochemical planes show lunasin (red), αVβ3 integrin, caveolin-1 or clathrin (green) and nuclei (blue) (**Figure 6A**). Macrophages were pretreated for 3 min with their respective endocytic inhibitors. Expression of αVβ3 integrin, caveolin-1 or clathrin (**E–H**) after treatment with lunasin and their specific inhibitors, respectively. Compared to the control (**A, I**), pretreatment with nystatin (54 µM, caveolae-mediated endocytic inhibitor) reduced lunasin internalization (**B, J**), while treatment with amantadine (1 mM, clathrin-mediated endocytic inhibitor) and amiloride (1 mM, macropinocytosis inhibitor) abolished lunasin uptake by macrophages (**C, J** and **D, L**). The effect of inhibitors on the endocytosis of lunasin (**Figure 6B**). In Figure 6B, the data represent the average of triplicates or quadruplicates ± SE. Four independent fields of view from three independent replicates of cells were merged together per treatment group. Means with different letters are significantly different from each other (n = 3, p<0.05). Bar  = 10 µm.

**Figure 6A** indicates that treatment with nystatin (a caveolae-mediated endocytosis inhibitor) reduced lunasin uptake by 60.9% (**Figure 6A** **, panel B, Video S3**) relative to the control (**Figure 6A** **, panel A**). Treatment with amantadine (a clathrin-mediated endocytosis inhibitor) and amiloride (a macropinocytosis inhibitor) reduced lunasin endocytosis by 97.7 and 99.5%, respectively (**Figure 6A** **, panels C, D, Videos S4, S5**). The green channel displays the immunofluorescence of the endocytic membrane protein that is associated with each treatment group: PBS control (**Figure 6A** **, panel E, αVβ3**), nystatin (**Figure 6A** **, panel F; caveolin-1**), amantadine (**Figure 6A** **, panel G; clathrin**) and amiloride (**Figure 6A** **, panel H; αVβ3**). The endocytic inhibitors did not significantly alter the expression of their respective target proteins. The results of merging all of the channels (**Figure 6A** **, panels I–L**) and their quantification indicates that lunasin endocytosis is largely associated with clathrin-coated structures and macropinosomes (**Figure 6B**).

### Lunasin elicited a transient reduction in the levels of free intracellular Ca^2+^ that was upregulated by LPS, whereas echistatin prevented the modulation of Ca^2+^ by lunasin

**Figure 7:**
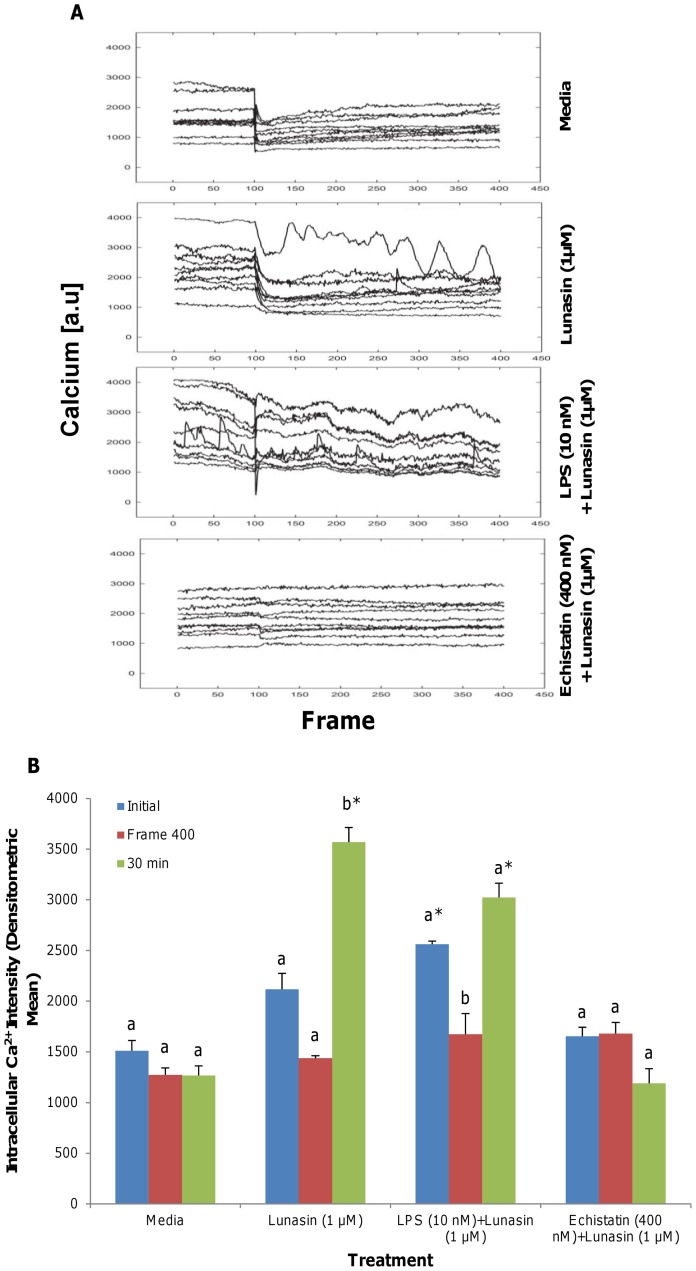
Lunasin transiently reduced free intracellular Ca^2+^ that was upregulated by LPS. Macrophages imaged with live-cell scanning confocal microscopy for intracellular free Ca^2+^. The median optical planes of individual cells were monitored for up to 400 frames (6.67 min) for the levels of free intracellular Ca^2+^ upon lunasin treatment (**Figure 7A**). Induction with LPS (10 nM) increased the levels of free intracellular Ca^2+^ in the macrophages compared to the unstimulated control, and lunasin transiently reduced this effect. After 30 min, resurgence in the levels of free intracellular Ca^2+^ was observed in the lunasin-treated cells. Echistatin, an αVβ3 inhibitor, prevented the modulation of the free intracellular Ca^2+^ by lunasin. Quantification of the intensity demonstrates the effect of lunasin on the intracellular Ca^2+^ levels over time (**Figure 7B**). Three independent fields of view from 10 independent replicates of cells were taken. Means within each treatment with different letters are significantly different from each other. An asterisk indicates that a treatment was significantly different from the media control (n = 3, p<0.05).

Live-cell laser-scanning confocal imaging of macrophages revealed increased levels of free intracellular Ca^2+^ in the macrophages that had been induced with the inflammatory ligand LPS (130 nM), compared to the unstimulated control (**Figure 7A** **, Video S6 a–c**). Lunasin (1 µM) treatment was initiated at 1.67 min (Frame 100) and transiently reduced the levels of Ca^2+^ by 34.6% over the course of 6.67 min (400 frames). Macrophages that had been pretreated with an αVβ3 inhibitor, echistatin (400 nM), did not exhibit changes in the initial levels of free intracellular Ca^2+^ and continued to remain unaffected after lunasin (1 µM) treatment. However, after 30 min of lunasin treatment, a resurgence of free intracellular Ca^2+^ was observed in the LPS-stimulated cells; levels increased by 44.6% compared to the levels that had been present at Frame 400 (p<0.05). In contrast, free intracellular Ca^2+^ levels remained completely unchanged in the lunasin-treated macrophages that had been pretreated with echistatin at all of the time points (p>0.05) (**Figure 7B** **, Video S6 d**).

## Discussion

The prevention of macrophage progression into a chronic pro-inflammatory state is essential to reducing the risk factors that are associated with CVD. The identification of the intracellular endosomal structures and effectors that could be involved in mediating the endocytosis of lunasin in macrophages facilitates the identification of the molecular targets of dietary anti-inflammatory RGD peptides. In this study, we report that lunasin internalization into macrophages is amplified when cells are in an inflammatory state and is primarily mediated by endocytic mechanisms that involve both clathrin-coated vesicles and macropinosomes.

The initial upregulation of the basal expression of the αVβ3 integrin at the membrane, due to treatment with the inflammatory ligands LPS, vitronectin, or the two molecules in combination, was subsequently attenuated upon lunasin treatment. Thus, lunasin endocytosis may be associated with integrin-mediated pathways and possesses the capacity to modulate αVβ3 integrin expression, even in the presence of potent agonists of inflammation, such as LPS or vitronectin. Lunasin uptake and αVβ3 integrin expression at the membrane increased most substantially in the cells that were stimulated with the combination of both LPS and vitronectin. Exposure to an oxidized low-density lipoprotein, which is a risk factor that has been strongly associated with the pathogenesis of atherosclerosis, upregulates αVβ3 integrin expression in human macrophages [13]. Moreover, synthetic αVβ3 antagonists and monoclonal antibodies directed against the integrin inhibited the development of atherosclerotic lesions in pigs that were fed a high-fat diet [14], [15], indicating that abnormal αVβ3 integrin expression is an important target to reduce these lesions. Induction with inflammatory agents and the subsequent upregulation of the αVβ3 integrin increased the number of receptor sites that were available for lunasin binding. The endocytosis of lunasin into the intracellular space facilitated the reduction in the number of available integrin receptor sites at the plasma membrane, suggesting the potential for lunasin in the prevention of inflammation associated with uncontrolled integrin signaling and the development of CVD.

The intracellular accumulation of lunasin localized within aggregates of clathrin-coated structures suggests that lunasin and these structures directly interact at specific regions. RGD peptides that had been conjugated to micelles were reported to internalize with αVβ3 integrin through clathrin-dependent endocytosis [12]. The increased recruitment of clathrin-coated vesicles to regions containing high concentrations of lunasin potentially indicates that the endocytosis of lunasin is mediated through the internalization of αVβ3 integrin via clathrin-coated vesicles. Two-dimensional median optical planes of nuclei exhibited punctate localization of lunasin within the nucleolar region. The endocytosis of cell-penetrating RGD peptides may cause them to target and sequester nucleoporin, which is a key component of the nuclear pore complex, and facilitate their entry into the nucleus [16]. Lunasin has been previously reported to translocate into the nuclei of cells within 24–72 h [17], [18]. Thus, the aggregation of the clathrin-coated structures and the punctate localization of lunasin at these intracellular sites could indicate a novel pathway for lunasin endocytosis and internalization into the nucleus via nucleolar sequestration.

Clathrin remained constitutively expressed at the membrane in stimulated macrophages upon lunasin treatment, suggesting that prior occupancy of the endocytic routes by other ligands affected lunasin internalization. Induction with LPS significantly reduced the basal expression of clathrin at the membrane, while vitronectin induction elicited no effect. However, subsequent treatment of the cells with exogenous lunasin increased clathrin levels at the membrane. This effect can be attributed to the fact that the endocytosis of LPS is dependent upon clathrin and early/sorting endosomes [19]. Thus, LPS may follow the same internalization route as lunasin, through clathrin-coated structures. In contrast, as an endogenous ligand of the αVβ3 integrin receptor, vitronectin binds to the available integrin receptors and may compete with lunasin. Thus, occupancy of the αVβ3 integrin receptor with vitronectin could impede the signal to increase clathrin expression upon lunasin treatment, which provides further evidence that lunasin directly binds to the αVβ3 integrin.

Lunasin promoted the recruitment of caveolin-1 to the plasma membrane surface, potentially through integrin-mediated signaling. Integrin signaling can facilitate the exocytosis of caveolin-1 in intracellular vesicles and the subsequent translocation of caveolin-1 to the surface of the plasma membrane [20]. Therefore, lunasin may activate this specific integrin-mediated signaling cascade and stimulate the release of caveolin-1 from the intracellular vesicles to the plasma membrane. Caveolin-1 is associated with integrin-mediated growth-limiting pathways and the deletion of this protein dissociates integrins from growth-regulatory pathways, resulting in aberrant, anchorage-independent growth [21]. Therefore, lunasin-mediated translocation of caveolin-1 proteins to the membrane surface could prevent abnormal macrophage proliferation and differentiation.

Brefeldin A abolished the ability of lunasin to be internalized by the cell, suggesting that BFA-dependent intracellular protein-trafficking pathways are essential for the endocytosis of lunasin. The endocytosis of RGD peptides can be regulated by integrin-mediated pathways that involve clathrin or caveolae. BFA disrupts protein trafficking from the Golgi–endoplasmic reticulum network, as well as the recycling of endocytic vesicles. As a result, newly synthesized proteins in the ER, such as clathrin, may remain anchored to the ER for as long as the secretory pathway of proteins is blocked [22]. Pretreatment with BFA may disrupt the integrin-recycling pathway, which may account for the constitutive expression of αVβ3 integrin at the membrane in the BFA-pretreated macrophages. By 6 h after BFA pretreatment, the reversible effect of BFA on intracellular protein trafficking appears to have been mitigated, possibly due to the endogenous lysosomal degradation of the BFA, resulting in resurgence in integrin expression. These results suggest that the internalization of lunasin is regulated by αVβ3 integrin-dependent endocytic proteins within the secretory pathway.

Lunasin endocytosis was abolished upon pretreatment with amantadine and amiloride, potentially indicating that its internalization is mediated primarily through clathrin-coated structures and is associated with macropinosomes. Inhibition of the caveolae-mediated pathway of endocytosis did not prevent lunasin internalization, demonstrating that, although caveolin-1 may be involved, this route is not the primary method of lunasin cell entry. The αVβ3 integrin has been reported to be internalized through both the clathrin- and caveolae-dependent pathways as part of the regulation of integrin turnover. It has been shown that a multimeric RGD peptide mediated the internalization of the αVβ3 integrin through clathrin-coated structures, and the endocytosis of this peptide was inhibited by a blockage in the clathrin-mediated endocytic pathway [23]. Therefore, the RGD cell-adhesion motif may play a role in integrin-dependent endocytosis and contribute to the capacity of lunasin to utilize multiple endocytic routes to gain access to the intracellular space.

Lunasin treatment elicited a temporal decrease in the levels of free intracellular Ca^2+^ that had been upregulated by the LPS stimulation, suggesting that lunasin transiently modulates divalent cation levels. Multiple cation-binding sites located within the integrin structure are involved in the regulation of the ligand-binding affinity of the receptor [24]. The presence of Ca^2+^ is associated with the stabilization of integrins in the low-affinity state, while divalent cations such as Mn^2+^ facilitate the activation of integrins for ligand-binding [25]. Co-immunoprecipitation experiments using whole-cell lysates from lunasin-treated macrophages demonstrated that lunasin directly interacts with the αVβ3 receptor, and LC/MS/MS was used to verify its identity within the integrin complex [7]. Therefore, the transient reduction in Ca^2+^ levels that is observed may be attributed to the binding of lunasin to the αVβ3 integrin, which in turn, activates the receptor from its low-affinity (high Ca^2+^) to its high-affinity state (low Ca^2+^). This series of events may facilitate ligand-binding and the observed endocytosis of lunasin. Moreover, the many aspartic acid residues of lunasin may contribute to its potential capacity to quench free intracellular Ca^2+^ through ionic interactions. Blockage of the αVβ3 integrin with RGD peptide and the inhibitor echistatin prevented the rise of Ca^2+^ levels when cells were subsequently exposed to the vitronectin ligand [26]. Pretreatment with echistatin completely prevented the modulation of Ca^2+^ levels that occurred with lunasin treatment in the absence of echistatin, further suggesting that Ca^2+^ signaling is inhibited by the saturation of αVβ3 integrin receptors.

Lunasin uptake and function in tissue-resident macrophages will be addressed in future work, since some evidence points to a specific cellular identity of these macrophages [27]–[29], which are probably distinct from LPS-elicited monocyte-derived macrophages.

## Conclusions

Taken together, these results indicate that lunasin endocytosis in human macrophages is amplified in inflammatory conditions and is primarily mediated by the endocytic pathways that are associated with integrin signaling, clathrin-coated vesicles and macropinosomes. The identification of the effectors that are involved in the mediation of lunasin endocytosis in human macrophages provides direct evidence for potential molecular targets of dietary RGD peptides and sheds light on the possible mechanisms by which these peptides may reduce inflammation and the risk of developing cardiovascular disease in humans.

## Supporting Information

Video S1 **Movie showing a 2D image stack of macrophages demonstrating the localization of lunasin (red), clathrin (green) and nuclei (blue) channels.** Lunasin is internalized in intracellular vesicles and localized inside large aggregates of clathrin-coated structures.
(MPG)

Video S2 **Movie showing a 2D image stack demonstrating the immunocytochemical localization of lunasin (red), αVβ3 integrin (green) and nuclei (blue) in macrophages that had been pretreated with brefeldin A (BFA, 71 µM) for 6 h.** BFA pretreatment inhibited the endocytosis of lunasin by the cell.
(MPG)

Video S3 **Movie showing a 2D image stack of macrophages that had been pretreated with nystatin (54 µM, caveolae-mediated endocytic inhibitor) along the Z axis for lunasin (red), caveolin-1 (green) and nuclei (blue).** Nystatin reduced lunasin internalization.
(MPG)

Video S4 **Movie showing a 2D image stack of macrophages that had been pretreated with amantadine (1 mM, clathrin-mediated endocytic inhibitor) along the Z axis for lunasin (red), clathrin (green) and nuclei (blue).** Amantadine abolished the endocytosis of lunasin.
(MPG)

Video S5 **Movie showing a 2D image stack of macrophages that had been pretreated with amiloride (1 mM, macropinocytosis inhibitor) along the Z axis for lunasin (red), αVβ3 (green) and nuclei (blue).** Amiloride inhibited the internalization of lunasin.
(MPG)

Video S6 **Movies showing 2D frames of macrophages that were imaged with live-cell scanning confocal microscopy for the free intracellular Ca^2+^ levels (pseudocolor) upon lunasin treatment.** a) culture media control; b) lunasin treatment; c) LPS-stimulated cells treated with lunasin; d) cells pretreated with echistatin and treated with lunasin. Lunasin transiently reduced the levels of free intracellular Ca^2+^, while echistatin prevented the modulation of calcium levels.
(MP4)
